# Comparison of the complications of traditional 12 cores transrectal prostate biopsy with image fusion guided transperineal prostate biopsy

**DOI:** 10.1186/s12894-016-0185-z

**Published:** 2016-11-17

**Authors:** Haifeng Huang, Wei Wang, Tingsheng Lin, Qing Zhang, Xiaozhi Zhao, Huibo Lian, Hongqian Guo

**Affiliations:** 1 Department of Urology, Affiliated Drum Tower Hospital, Medical School of Nanjing University, Nanjing, 210008 China; 2 Institute of Urology, Nanjing University, Nanjing, 210008 China

**Keywords:** Prostate biopsy, Prostate cancer, Fusion image, Magnetic resonance imaging, Complications

## Abstract

**Background:**

To compare the complications of traditional transrectal (TR) prostate biopsy and image fusion guided transperineal (TP) prostate biopsy in our center.

**Methods:**

Two hundred and fourty-two patients who underwent prostate biopsy from August 2014 to January 2015were reviewed. Among them, 144 patients underwent systematic 12-core transrectal ultrasonography (TRUS) guided prostate biopsy (TR approach) while 98 patients underwent free-hand transperineal targeted biopsy with TRUS and multi-parameter magnetic resonance imaging (mpMRI) fusion images (TP approach). The complications of the two groups were presented and a simple statistical analysis was performed to compare the two groups.

**Results:**

The cohort of our study include242 patients, including 144 patients underwent TR biopsies while 98 patients underwentTP biopsies. There was no significant difference of major complications, including sepsis, bleeding and other complication requiring admissionbetween the two groups (*P* > 0.05). The incidence rate of infection and rectal bleeding in TR was much higher than TP (*p* < 0.05), but the incidence rate of perineal swelling in TP was much higher than TR (*p* < 0.05). There were no significant differences of minor complications including hematuria, lower urinary tract symptoms (LUTS), dysuria, and acuteurinary retention between the two groups (*p* > 0.05).

**Conclusion:**

The present study supports the safety of both techniques. Free-handTP targeted prostate biopsy with real-time fusion imaging of mpMRI and TR ultrasound is a good approach for prostate biopsy.

**Electronic supplementary material:**

The online version of this article (doi:10.1186/s12894-016-0185-z) contains supplementary material, which is available to authorized users.

## Background

Biopsies of the prostate have been used to diagnose prostate cancer since the beginning of the last century [1]. The field of prostate diagnostics, especially biopsy techniques develops rapidly [2]. Transrectal ultrasonography (TRUS) guided prostate biopsy, which isperformed with a core biopsy needle passing through the rectum, was first applied for the biopsy of prostate in 1968 [3]. Since the introduction of the systematic 12-core transrectal prostate biopsy guided by TRUS, it has become awidely accepted, routinely performed technique for prostate cancer detection [4]. The transperineal prostate biopsy, which is performed with the core biopsy needle passing through the skin of the perineum, is far less common compared with transrectal biopsy [5].

Several studies have demonstrated that the transrectal technique is a faster and convenient approach for prostate biopsy. Though the minor complications rate of hematuria, rectal bleeding, hematospermia, vasovagal episodes, infection was reported to be similar in these two techniques [6], transrectal prostate biopsy had more major complicationse.g. sepsis, bleeding or other complications requiring admission compared with the transperineal biopsy [7, 8]. More importantly, an increasing risk of septic shock was reported in the transrectal biopsy, which might be life-threatening [9, 10].

Recently, transperineal prostate biopsy has been becoming an increasingly popular approach for accurate diagnosis and risk stratification of prostate cancer [11]. MRI/US-fusion-guided biopsyis a potential approach to offer improved diagnostic information over systematic 12-core transrectal prostate biopsy guided by TURS alone [12, 13]. We have previously described free-hand transperineal targeted biopsy guided by TRUS and mpMRI fusion images [14]. The targeted biopsy is a precise, faster and more accessible technique for prostate biopsy compared to systematic biopsy. In the present study, we reported the complications of transperineal targeted biopsy guided by TRUS-mpMRI fusion images and traditional 12 cores systematic transrectal biopsy guided by TURS in our center.

## Methods

The study protocol was approved by the local institutional review board at Nanjing University (NJU201500987), and informed written consent was received from patients, including acquiring essential medical images for publication. Research was carried out in compliance with the Helsinki Declaration.

### Patients

#### Procedures

The present study was performed by reviewing a total of 262 patients who underwent prostate biopsy from August 2014 to January 2015. At the time of analysis, the database contained 262 patients of whom 20 were excluded because of <10 cores taken in systematic biopsies. Thus, 242 patients were available for analysis. These patients with PSA level greater than 4.0 ng/mL underwent mpMRI prospectively. All these patients were assessed with prostate mpMRI in the radiology department of our hospital, and 98 patients had at least one suspicious areas in mpMRI images who underwent free-hand transperineal targeted biopsy with TRUS and multi-parameter magnetic resonance imaging (mpMRI) fusion images (TP approach). The other 144 patients underwent systematic 12-core Transrectal Ultrasonography (TRUS) guided prostate biopsy (TR approach). No patients had any previous history of prostate biopsy. All patients had given informed consent. Patient demographics including age, digital rectal examination (DRE) findings (recorded as benign, suspicious, malignant or unknown), Body Mass Index (BMI, kg/m2), prostate volume, serum total prostate specific antigen (PSA), biopsy technique (transperineal or transrectal), total number of cores taken, number of cores positive for prostate adenocarcinoma from histology, Gleason grade and score, positive biopsies, complications (The major complications included sepsis and severe hematuria, the minor complications included minor hematuria, LUTS (lower urinary tract symptoms), dysuria, acute urinary retention, infection, rectal bleeding, perineal swelling) and treatments of the study cohort are shown in Table 1. In our study, rectal bleeding was defined as the passage of bright red blood rectally > 12 h after biopsy, or any bleed requiring active management irrespective of the time of occurrence. Major hameaturia was defined as obvious gross hematuria with or without blood clot, minor hameaturia was defined as no obvious gross hematuria and was confirmed microscopic hematuria.

**Table 1:** Characteristics of patients

Parameter	TR	TP	*P*
Patients (n)	144	98	--
Age, yr (range)	65.61 ± 11.21 (51~86)	63.40 ± 9.81 (50~77)	0.107
BMI, kg/m^2^ (range)	27.81 ± 4.86 (23.5~34.7)	28.60 ± 5.17 (22.4~35.1)	0.221
PSA, ng/ml (range)	11.23 ± 6.82 (6.3~46.7)	9.61 ± 7.94 (4.6~36.6)	0.104
Prostate volume, ml (range)	32.62 ± 9.11 (20.5~67.8)	34.21 ± 6.42 (23.5~59.8)	0.110
Suspicious DRE findings (rate)	9 (6.3%)	6 (6.1%)	0.968
biopsy time, min (range)	11.55 ± 6.71 (10~26)	16.61 ± 7.82 (12~39)	<0.001
biopsy number (range)	12	15.16 (13~17)	<0.001
Gleason score (range)	6.89 ± 0.75 (6~9)	7.10±0.89 (6~9)	0.057
Follow up, months (range)	7.51 ± 4.26 (1~12)	8.12±5.15 (1.5~13)	0.337

All prostate biopsies were performed by an experienced urologist. (W. W). We used the cefuroxime sodium (1.5g, intravenous drip) for anti-infective prophylactic therapy. Systematic 12-core prostate biopsy were performed under transrectal ultrasound guidance, a standard of twelve cores was taken using TR approach. TR approach were performed with the patient in the left decubitus position, using an 18-G automatic biopsy gun with a specimen size of 22 mm (Bard Magnum; Bard Medical, Covington, GA, USA), 20 ml of Lidocaine gel was introduced intra-rectally 15 min before the procedure, the sextant biopsies were taken laterally in the prostate from the base, midline and apex, 3 cores were taken from each side from the far lateral areas of the prostate at the base, midline and near the apex. TP approach was performed according to our previous study. Multi-parametric MRI examination and TRUS-mpMRI image fusion were shown in Additional file 1. Biopsy protocol: The biopsy started with target biopsy to the center of cancer-suspicious lesions without the guide of template using the free-hand transperineal technique, and then, standard 12-core stand biopsy (blinded to the MRI target lesions) was carried out in all patients. Lesions suspicious of cancer identified on MRI were semi automatically displayed on the real-time TRUS image [13]. All target lesions were sampled once in both axial and sagittal planes, with at least two core biopsies per target. An 18-G automatic biopsy gun with a specimen size of 22 mm (Bard Magnum; Bard Medical, Covington, GA, USA) was used to take biopsy cores. All patients underwent general anesthesia using a larynx mask during the biopsy procedure.

### Statistical analysis

Data are presented as the Mean ± SD. Statistical analysis involved use of SPSS 17.0 (SPSS Inc, Chicago, IL). Between-group comparisons involved *t* test and chi-square test. *P*<0.05 was considered statistically significant.

## Results

Patients demographics are shown in Table 1. The patients were divided into two groups based on the different prostate biopsy techniques. A total of 242 patients were included in the study. Among them, 144 men underwent transrectal biopsies while 98 men underwent transperineal biopsies. The mean age of the TR biopsies and TP biopsies was 65.61 ± 11.2 years (range 51–86 years) and 63.40 ± 9.81 years (range 50–77 years)respectively. The mean BMI of TR and TP was 27.81 ± 4.86 kg/m^2^ (range 23.5 ~ 34.7 kg/m^2^) and 28.60 ± 5.17 kg/m^2^ (range 22.4 ~ 35.1 kg/m^2^) respectively. The mean preoperative PSA value of TR and TP was 11.23 ± 6.82 ng/mL (range 6.3 ~ 46.7 ng/mL) and 9.61 ± 7.94 ng/mL (range 4.6 ~ 36.6 ng/mL) respectively. The mean preoperative prostate volume of TR and TP, calculated using the ellipsoid formula, was 32.62 ± 9.11 mL (range 20.5 ~ 67.8 mL) and 34.21 ± 6.42 mL (range 23.5 ~ 59.8 mL), respectively. Nine patients in TR (6.3%) had a recognized palpable nodule in the prostate based on digital rectal examination (DRE), and six patients in TP (6.1%) had suspicious DRE findings. The mean biopsy time of TR was 11.55 ± 6.71 min (range 10–26 min). The mean biopsy time of TP, including the MRI-TRUS fusion time and needle puncture time without anesthesia, was 16.61 ± 7.82 min (range 12–39 min). The biopsy time of TR was shorter than TP (*p* < 0.001). The mean Gleason score of TR was 6.89 ± 0.75 (range 6 ~ 9) whilethe mean Gleason score of TP was 7.10 ± 0.89 (range 6 ~ 9). The mean follow-up of TR was 7.51 ± 4.26 months (range 1 ~ 12 months) while the mean follow-up of TP was 8.12 ± 5.15 months (range 1.5 ~ 13 months). There were no significant differences between TR and TP in age, BMI, PSA, prostate volume, suspicious DRE findings, Gleason score, during the follow-up period (*p* > 0.05).

The comparative pathological results of prostate biopsies in TR and TP are shown in Table 2. The TR and TP detected prostate cancer (PCa) in 57 (39.6%) and 51 (52.0%) patients, respectively. The TR detected High-grade PIN, Low-grade prostatic intraepithelial neoplasia (PIN) in 10 (6.9%) and 7 (4.9%) patients, respectively. The TP detected High-grade PIN, Low-grade PIN in 5 (5.1%) and 6 (6.1%) patients, respectively. The TR and TP detected benign prostate hyperplasia (BPH) in 59 (40.9%) and 32 (32.7%) patients, respectively. The TR and TP detected chronic prostatit is in 11 (7.6%) and 4 (4.1%) patients, respectively.

**Table 2:** Comparison of the pathological results TR biopsy and TP biopsy

Group	PCa *n* (%)	High-grade PIN *n* (%)	Low-grade PIN *n* (%)	BPH *n* (%)	Chronic prostatitis *n* (%)	Total *n* (%)
TR	57 (39.6)	10 (6.9)	7 (4.9)	59 (40.9)	11 (7.6)	144
TP	51 (52.0)	5 (5.1)	6 (6.1)	32 (32.7)	4 (4.1)	98
*P* value	0.055	0.559	0.669	0.189	0.259	/

*PIN* prostate intraepithelial neoplasm

Comparison of the positive (detected PCa) and negative (undetected PCa) results of TR and TP according to PSA classification is shown in Table 3. In patients who’s PSA level lower than 10ng/ml, The TR and TP detected PCain 8 and 4patients, respectively. (*p* = 0.401). In patients who’sPSA between 10 to 20ng/ml, The TR and TP detected PCain13 patients, respectively (*p* = 0.895). In patients who’sPSA higher than 20ng/ml, The TR and TP detected PCain36 and 40 patients, respectively (*p* = 0.029). The PCa detection rate of TP was much higher than TR in patients who’sPSA higher than 20ng/ml (*p* < 0.05).

**Table 3:** Comparison of the positive and negative results of TR and TP

PSA	Positive, *n*		Negative, *n*		*P* value
	TR	TP	TR	TP	
<10	8	4	37	32	0.401
10~20	13	7	26	11	0.895
>20	36	40	20	8	0.029

Complications rates of the two groups were recorded and the results are presented in Table 4. The major complications included sepsis and severe hematuria. The minor complications included minor hematuria, lower urinary tract symptoms (LUTS), dysuria, acute urinary retention, infection, rectal bleeding, perineal swelling. In the TR group, one patient (0.7%) had sepsis and treated with admitted intravenous (IV) antibiotics. Two patients (1.38%) had severe hematuria. In the TP group, no patient had major complications. However, there was no significant difference between two groups of major complications (*P* > 0.05). There were significant differences between the two groups of minor complications in infection, rectal bleeding, and perineal swelling. As shown in Table 4,there were 16 patients in TR (11.11%) and 3 patients in TP (3.06%) with infection.2 patients in TR (1.4%) and no patients in TP (0%) had rectal bleeding. 3 patients in TR (2.08%) and 10 patients in TP (10.2%) had perineal swelling. The rate of infection and rectal bleeding in TR were much higher than TP (*p* < 0.05) However, the rate of perineal swelling in TP was much higher than TR (*p* < 0.05). There were no significant differences of minor complications including minor hematuria, LUTS dysuria, acute urinary retention between thetwo groups (*p* > 0.05), in spite of more biopsy cores in TP biopsy.

**Table 4:** Comparison of the incidence of complications of TR and TP biopsy

Group	TR (*n*, %)	TP (*n*, %)	*P* value
Major			
sepsis	1 (0.70)	0 (0.00)	0.408
Severe haematuria	2 (1.38)	0 (0.00)	0.241
Minor			
Minor hematuria	45 (31.25)	35 (35.71)	0.468
LUTS	24 (16.67)	25 (25.51)	0.091
Dysuria	9 (6.25)	11 (11.22)	0.168
Acute urinary retention	13 (9.03)	10 (10.20)	0.759
Infection	16 (11.11)	3 (3.06)	0.022
Rectal bleeding	2 (1.4)	0 (0)	<0.001
Perineal swelling	3 (2.08)	10 (10.20)	0.006

Total complications in each patient of TR and TP were shown in Table 5. There were 18 patients (12.5%) without any complication after biopsy in TR and 29 patients (29.59%) without any complication after biopsy in TP. There were more patients without any complications in TP group than that in TR (*p* < 0.001). 43 patients (29.86%) with one complication were detected in TR and 41 patients (41.83%) in TP. 45 patients (31.25%) with two complications after biopsy were found in TR group and 21 patients (21.42%) in TP.34 patients (23.61%) were found with more than three complications after biopsy in TR and 7 patients (7.14%) in TP. The rate of multiple complications after biopsy in TR were much higher than TP (*p* < 0.001).

**Table 5:** Total complications in each patient of TR and TP

Total complications	TR (*n*, %)	TP (*n*, %)	*P* value
0	18 (12.5)	29 (29.59)	<0.001
1	43 (29.86)	41 (41.83)	0.055
2	45 (31.25)	21 (21.42)	0.092
≥3	34 (23.61)	7 (7.14)	<0.001

## Discussion

The present results showed that cancer detection rate of TP group were higher than TR group (Table 2). This might be due tothat the TP group had at least one suspicious area in mp MRI images and included at least one targeted biopsy, but the TP group had notargeted biopsy.

Complication rate of prostate biopsies varies widely in medical literature. Several studies reveal major complications (sepsis, bleeding or other complication requiring admission) rates of around 1–2% in transrectal prostate biopsy, with the following rates of minor complications: hematuria 10–84%; rectal bleeding 1.3–45%; hematospermia 1.1–93%; vasovagal episodes 0–5%; infective complications 0–6.3% [7, 15]. Studies also reveal similar wide-ranging rates of minor complications in transperineal biopsies. Recently, a systematic review showed that the rate of sepsis after TRUS biopsy is as high as 5%, and appears to be rising with increasing rates of multi-resistant bacteria found in rectal flora. However, the rate of sepsis from published series of TP biopsy approached zero [10], andour results was consistent with the conclusions from the literature.

In our study, the major complications rate following TR was 2%, whereas no patient had a major complication following TP (Table 4). However, there was no significant difference between the rate of major complications in the two groups (*P* > 0.05). Of note, there was one case of sepsis in the TR approach, and cured by admitted intravenous drip antibiotics. Visible hematuria following prostate biopsy is common, with reported rates of 10–84% [15]. Bleeding post procedure is usually self-limiting and rarely life threatening. Two patients had severe hematuria and cured by continuous bladder irrigation. Mortality after prostate biopsy is extremely rare, and most reported deaths are the result of septic shock. In fact, there was no patient died after prostate biopsy in the present study.

Minor complication rates in both techniques were comparable. Infection is a well-established risk of prostate biopsy. Various strategies to reduce infectious complications have been explored, as were recently reviewed [9]. Recently, several studies have shifted from the transrectal to the transperineal technique with anecdotal reports of very low rates of sepsis, even without the use of prophylactic antibiotics [10, 16]. In this study, we also found that the rate of infection in TR was significantly higher than TP (*p* < 0.05). The rate of infection and rectal bleeding in TR were much higher than TP (*p* < 0.05), while the rate of perineal swelling in TP was much higher than TR (*p* < 0.05). This might be due to the different approaches since more biopsy cores were taken in TP procedure. Bleeding is the most frequently reported complication after biopsy, but it is usually minor and resolves spontaneously. The present study was unable to prove any significant difference of minor complications including minor hematuria, LUTS, dysuria, acute urinary retention between the two groups (*p* > 0.05).

The TR approach is a much easier procedure and can prevent patients’ discomfort by using only local anesthesia. This changed after several reports of fatalities following septic complications of transrectal biopsy [17]. Image fusion guided TP biopsy is increasingly popular as a mean for accurate diagnosis and risk stratification [11, 18]. An increasing number of studies have showed that MRI/US image-fusion guided TP is an effective and accurate method for prostate cancer diagnosis. Our study also showed a higher prevalence of MRI/US image-fusion guided TP proven cancers in suspicious areas, which can result in better prostate cancer characterization via precise localization, prediction of Gleason grade, and more accurate cancer core length. Moreover, the incidence rate of infection and rectal bleeding after biopsy in TP was much less than TR.

There were some limitations in the present study. The first is the retrospective study design was absence of randomization. Second, there was inadequate sample size in this study. Third, the procedure of TR with local anesthesia was simplicity, but the procedure of TP with general anesthesia was complexity. It is unknown whether this difference has clinical significance, and how it affects the complication rates. Further prospective multi-center randomized controlled trials will be conducted.

## Conclusion

The present study supports the safety of both trans-rectal prostate biopsy and free-hand transperineal targeted prostate biopsy with real-time fusion imaging of mpMRI/US in the diagnosis of prostate cancer. Transperineal prostate biopsy is safe with no cases of sepsis recorded, and the rate of infection and rectal bleeding in TP is much lower than TR, in spite of more biopsy cores in TP biopsy. This suggests that free-hand transperineal targeted prostate biopsy with real-time fusion imaging of mpMRI/US can be a good approach for prostate biopsy.

## Additional file

Additional file 1: Multiparametric MRI Examination and Analysis. (DOCX 14 kb)

## Abbreviations

DRE: Digital rectal examination
mpMRI: Multi-parameter magnetic resonance imaging
TP: Transperineal
TR: Transrectal
TRUS: Transrectal Ultrasonography
