# Supplementary material for: Comparison of the complications of traditional 12 cores transrectal prostate biopsy with image fusion guided transperineal prostate biopsy
Source: BMC Urol. 2016 Nov 17;16:68. doi: 10.1186/s12894-016-0185-z (PMC5114768; doi:10.1186/s12894-016-0185-z)
Supplement: Additional file 1: — Multiparametric MRI Examination and Analysis. (DOCX 14 kb) [file 12894_2016_185_MOESM1_ESM.docx]

Multiparametric MRI Examination and Analysis
Multiparametric prostate MRI was performed with a 3.0-TMR scanner (Achieva 3.0T TX dual-source parallel RF excitation and transmission technology, Philips Medical Systems, The Netherlands) by using a 32-channel phased array coil. Transverse/coronal/sagittal (18 slices, thickness3 mm/gap 0.5 mm, TR 3744 ms, TE 120 ms, number of signals acquired 2, resolution 1.49 mm × 1.51 mm) T2-weighted turbo spin-echo (TSE) images were acquired. Diffusion-weighted imaging, spin-echo–echo-planar images (18 slices, thickness 6 mm, intersection gap 1 mm, TR 925/TE 41 ms, number of signals acquired 1, resolution 3 mm × 3 mm, b-factor 0/800 s/mm2) were acquired. And Thigh-resolution isotropic volume with fat suppression after gadolinium injection was employed for dynamic contrast-enhanced images (133 slices, thickness 3 mm, no intersection gap, TR 3.1/TE 1.46 ms, number of signals acquired 1, resolution 1.49 mm × 1.51 mm, dynamic scan time 00:06.9). Mappings of the apparent diffusion coeffcient (ADC) were generated from b 0 and b 1000 images of DWI using the Philips WorkStation software (Extended Workspace, EWS).All MRI scans were reviewed by an experienced radiologist (B.Z.) with no prior clinical information. Suspicious areas, so-called region of interest (ROI), were defined, and
the radiologist provided a likelihood score that clinically significant cancer would be present for each ROI from 2 to5 on the PI-RAD classifcation based on a Likert scale according to the European Society of Urogenital Radiology prostate MR guidelines 2012: 1, most probably benign; 2, probably benign; 3, indeterminate; 4, probably malignant; and 5, highly suspicious of malignancy.

mpMRI and TRUS image fusion
All biopsies were conducted with an mpMRI–TRUS biopsy system (RVS®, Real-time Virtual Sonography, Hitachi Medical Corporation, Tokyo, Japan) that provides real time fusion of TRUS images and MR images to guide the biopsy needles using a transperineal approach. The procedure of MRI fused to the real-time TRUS was as described previously. In brief, the documented lesions were marked first as ROI on morphological, high-resolution transversal TW TSE sequences, which were loaded into the biopsy system before. The magnetic field generator was placed near the patients’ body, and the magnetic position sensor was mounted on the ultrasound probe to be used for acquiring the TRUS images. The internal urethral orifice was used as the fducial landmark for registering the MRI and ultrasound images. The MR images reconstructed from the MRI volume data that corresponded to the US sagittal images were displayed adjacent to the US sagittal image on the same monitor. Morphological MRI data including the apparent ROIs were superimposed on the TRUS images

in real time to guide the biopsy needle. The system documented the positions of the acquired cores in the Digital Imaging and Communication in Medicine (DICOM) dataset of the morphological MRI with consecutive numbers.
